# A novel CD147 inhibitor, SP-8356, reduces neointimal hyperplasia and arterial stiffness in a rat model of partial carotid artery ligation

**DOI:** 10.1186/s12967-019-2024-y

**Published:** 2019-08-20

**Authors:** Kisoo Pahk, Hyojin Noh, Chanmin Joung, Mi Jang, Hwa Young Song, Kyung Won Kim, Kihoon Han, Jong-Ik Hwang, Sungeun Kim, Won-Ki Kim

**Affiliations:** 1 Institute for Inflammation Control, Korea University, Seoul, South Korea; 2 Department of Neuroscience, Korea University College of Medicine, 126-1, Anam-Dong 5-Ga, Seongbuk-Gu, Seoul, 136-705 South Korea; 3 Department of Nuclear Medicine, Korea University Anam Hospital, Seoul, South Korea; 4 Department of Radiology, Asan Medical Center, University of Ulsan College of Medicine, Seoul, South Korea; 5 Department of Biomedical Sciences, Korea University College of Medicine, Seoul, South Korea

**Keywords:** Atherosclerosis, Neointimal hyperplasia, Arterial stiffness, Vascular smooth muscle cell, CD147, Matrix metalloproteinase, MMP-9

## Abstract

**Background:**

Neointimal hyperplasia and its related arterial stiffness are the crucial pathophysiological features in atherosclerosis and in-stent restenosis. Cluster of differentiation 147 (CD147), a member of the immunoglobulin super family that induces the expression of matrix metalloproteinase-9 (MMP-9) by dimerization, may play important roles in neointimal hyperplasia and may therefore be an effective target for the treatment of this condition. Here, we investigated whether a novel CD147 inhibitor SP-8356 ((1S,5R)-4-(3,4-dihydroxy-5-methoxystyryl)-6,6-dimethylbicyclo[3.1.1]hept-3-en-2-one) reduces neointimal hyperplasia and arterial stiffness in a rat model of partial carotid artery ligation.

**Methods:**

Neointimal hyperplasia was induced in Sprague–Dawley rats by partial ligation of the right carotid artery combined with a high fat diet and vitamin D injection. Rats were subdivided into vehicle, SP-8356 (50 mg/kg), and rosuvastatin (10 mg/kg) groups. The drugs were administrated via intraperitoneal injections for 4 weeks. The elasticity of blood vessels was assessed by measuring pulse wave velocity using Doppler ultrasonography before sacrifice. Histomolecular analysis was carried out on harvested carotid arteries.

**Results:**

SP-8356 significantly reduced MMP activity by inhibiting CD147 dimerization. SP-8356 reduced neointimal hyperplasia and prevented the deterioration of vascular elasticity. SP-8356 had a greater inhibitory effect on neointimal hyperplasia than did rosuvastatin. Furthermore, rosuvastatin did not improve vascular elasticity. SP-8356 increased the expression of smooth muscle myosin heavy chain (SM-MHC), but decreased the expression of collagen type III and MMP-9 in the neointimal region. In contrast to SP-8356, rosuvastatin did not alter the expression of SM-MHC or MMP-9.

**Conclusions:**

The ability of SP-8356 to reduce neointimal hyperplasia and improve arterial stiffness in affected carotid artery suggests that SP-8356 could be a promising therapeutic drug for vascular remodeling disorders involving neointimal hyperplasia and arterial stiffness.

## Background

Neointimal hyperplasia, defined as the thickening of the arterial intima with a narrowed arterial lumen space, is a key feature of early atherosclerotic lesions and in-stent restenosis [1, 2]. The key regulatory mechanism underlying neointimal hyperplasia is the phenotypic plasticity of vascular smooth muscle cells (VSMCs) [2]. In a normal physiologic state, most VSMCs in the blood vessels show contractile characteristics [2, 3]. However, exposure of the vessels to injury or inflammatory stimuli results in VSMC switching to a synthetic phenotype. These synthetic VSMCs migrate to the intima from the media and contribute to the formation of neointimal hyperplasia [4]. Furthermore, synthetic VSMCs in neointima accelerate lipid deposition and macrophage chemotaxis, leading to the progression of atherosclerosis [1, 5]. Thus, synthetic VSMCs not only build up neointima but may also promote plaque vulnerability.

Neointimal hyperplasia is composed of migrated synthetic VSMCs with lower amounts of contractile proteins and higher amounts of extracellular matrix (ECM) components [2]. These changes in vascular smooth muscle tone and ECM components contribute to arterial stiffening [6]. Arterial stiffness has been regarded as an important predictor of future cardiovascular events and all-cause mortality [7]. Therefore, attenuation of neointimal hyperplasia may be an important therapeutic target for patients with vascular remodeling disorders, including atherosclerosis and in-stent restenosis.

Matrix metalloproteinases (MMPs) have been regarded as a key player in VSMC migration [8, 9]. MMPs are proteolytic enzymes that cleave ECM and modulate chemokines. Chemokines, in turn, facilitate the easy migration of VSMCs to neointima and initiate synthetic VSMC activities [8]. Among the various MMPs, particularly, overexpression of MMP-9 is well known to enhance VSMC migration with synthetic properties [8, 9]. MMP expression can be induced by cluster of differentiation 147 (CD147), which is located on the VSMCs [10]. CD147, also known as extracellular MMP inducer (EMMPRIN), is a cell surface glycoprotein that induces MMP expression [11–13] by homophilic interactions such as dimerization [13, 14].

We recently reported that a series of (1S)-(-)-verbenone derivatives exhibited cytoprotective activities [15] and found that certain (1S)-(-)-verbenone derivatives in an in-house chemical library inhibited cerebral hemorrhage by inhibiting MMP activity (unpublished results). One of these (1S)-(-)-verbenone derivatives, (1S,5R)-4-(3,4-dihydroxy-5-methoxystyryl)-6,6-dimethylbicyclo[3.1.1]hept-3-en-2-one (SP-8356), was found most effective in reducing MMP activity by inhibiting CD147 dimerization. Recently, we and others reported that partial carotid artery ligation induced neointimal hyperplasia in rats fed a high fat diet [16, 17]. The present study investigated the ability of SP-8356 to inhibit VSMC migration and arterial stiffness in this rat model characterized by neointimal hyperplasia.

## Methods

### Non-denaturing sodium dodecyl sulfate–polyacrylamide gel electrophoresis (SDS-PAGE) assays

Recombinant human CD147 protein (5 μg/mL, ab155636, Abcam, Cambridge, MA, USA) was added to various concentrations of SP-8356 and mixed with sample buffer lacking SDS, followed by resolution by 10% SDS-PAGE without boiling. Anti-CD147 antibody (ab108317, Abcam, Cambridge, MA, USA) was used for immunoblotting. The concentration of recombinant human CD147 protein was determined following the previous study which reported the inhibitory effect of AC-73 on CD 147 dimerization [18].

### Surface plasmon resonance (SPR) assays

SPR assays were performed on an SR7500DC instrument (Reichert Inc., Buffalo, NY, USA) at 25 °C. Approximately 4000 resonance units (RU) of recombinant human CD147 (ab155636, Abcam), at a concentration of 3 μg/mL in 10 mmol/L sodium acetate (pH 4.5), were immobilized on CMDH chips containing gold (Reichert Inc., Buffalo, NY, USA), using the amine coupling kit supplied by the manufacturer. The analyte (SP-8356; 300.35 kDa) was dissolved in running buffer (1X PBS, 2% DMSO, pH 7.4) and injected over the flow cells at concentrations of 6.25, 12.5, 25, 50, 100, 200 and 400 μM, in that order, at a flow rate of 30 μL/min. As a control, 1.25–640 nM anti-CD147 antibody (ab108317, Abcam), from lowest to highest concentration, was injected over the flow cells to test its binding to immobilized CD147. The association and dissociation times were both 5 min. After each round, the surface of the sensor chip was regenerated by injection of NaOH (5–50 mM) for 1 min until the RU signal returned the baseline. An equilibrium dissociation rate constant (*K*d) was calculated from the kinetic rate constants by a simple 1:1 interaction model using Scrubber 2 software (Biologic Software, Campbell, Australia).

### Cell culture

The A10 vascular smooth muscle cell line (ATCC CRL 1476) was purchased from the American Type Culture Collection (ATCC, Manassas, VA, USA). The cells were cultured in Dulbecco’s modified eagle medium (DMEM; Welgene, Daegu, Korea) supplemented with 10% fetal bovine serum (FBS; HyClone, Logan, UT, USA), and 1% penicillin/streptomycin (HyClone) at 37 °C in a 5% CO_2_ humidified atmosphere. The contractile type of VSMC was induced by serum deprivation for 6 days [19].

### Immunocytochemistry

To assess whether SP-8356 affects VSCM phenotype modulation, induced contractile VSMCs were pre-treated with SP-8356 for 30 min and subsequently treated with recombinant human protein CD147 (5 μg/mL, ab155636, Abcam, Cambridge, MA, USA). After 24 h of incubation, cells were washed with phosphate-buffered saline and fixed with 4% paraformaldehyde. Cells were then permeabilized with 0.3% Triton X-100 and blocked for 30 min with 1% bovine serum albumin in PBST, followed by incubated at 4 °C with primary antibody to smooth muscle myosin heavy chain (SM-MHC) (1:200 dilution, ab683, Abcam, Cambridge, MA, USA) for overnight. Alexa Fluor 488-conjugated goat anti-mouse IgG (1:400 dilution, A28175, Invitrogen, Carlsbad, CA, USA) was used for secondary antibody and nuclei were stained with Hoechst 33,342 solution.

### Gelatin zymography

MMP activities of cultured VMSCs were evaluated by gelatin zymography. A10 VSMCs were treated for 24 h with SP-8356 in the presence of recombinant human protein CD147 (5 μg/mL, ab155636, Abcam, Cambridge, MA, USA). Conditioned media were collected and centrifuged to remove cellular debris and concentrated by Microcon centrifugal filtration (Millipore, Billerica, MA, USA). These samples were mixed with a non-reducing loading buffer without heating and loaded onto 10% SDS-PAGE gels containing 1 mg/mL gelatin (JT Baker Chemical Co., Phillipsburg, NJ, USA). Proteins were separated by electrophoresis at 125 V for 90 min. A MMP-9 recombinant protein (ab168863, Abcam, Cambridge, MA, USA) was loaded as a positive control. Following electrophoresis, the gels were rinsed twice for 30 min with Novex zymography renaturing buffer (Invitrogen, Carlsbad, CA, USA), incubated overnight with Zymogram developing buffer (Invitrogen), and stained with a Simply Blue Safe Stain (Invitrogen).

### Migration assay

VSMC migration assays were performed with 24-well Transwell plates and polycarbonate membranes (8-μm pore size, Costar, Corning, NY, USA) coated with Matrigel (Sigma, St Louis, MO, USA). The upper chambers were seeded with 1.5 × 10^5^ VSMCs in 200 μL serum-free DMEM. The Transwells were placed in 24-well culture dishes in 800 μL serum-free DMEM. After incubation for 12 h with SP-8356 in the presence of recombinant human CD147 (5 μg/mL, ab155636), the membranes were fixed and stained with a Hemacolor rapid staining kit (Merck, Darmstadt, Germany). The number of cells that had migrated to the lower chambers was counted (5 random fields/membrane) using an inverted microscope (Leica DM IL, Leica Microsystems, Wetzlar, Germany).

### Animals

Male Sprague–Dawley rats (7 weeks old, 200–250 g body weight) were purchased from Orient-Bio (Seongnam, Korea). All rats were housed under a 12-h light/dark cycle with ad libitum access to water and food.

### Induction of neointimal hyperplasia

After the rats were acclimated for 1 week, they were subjected to partial right carotid artery ligation, as described [17, 20]. In brief, anesthesia was initiated with 3.5% isoflurane in a 2:1 N_2_O/O_2_ mixture in a vented anesthesia chamber and sustained by inhalation through a nasal cone of 2 to 2.5% isoflurane in a 2:1 N_2_O/O_2_ mixture. The right external carotid artery, occipital artery and right internal carotid artery were ligated with 6-0 silk sutures, followed by intraperitoneal injection of vitamin D3 (6 × 10^5^ IU/kg) once daily for 2 days. Vitamin D3 is often used to stimulate VSMC proliferation and migration [21, 22]. All rats were fed with a commercially available high fat diet (D12336, Research Diets, NJ, USA) for 1 month (28 days).

### Drug treatment

The day after their last vitamin D injection, the rats were subdivided into three groups. The rats were injected intraperitoneally with vehicle (0.9% normal saline), rosuvastatin (10 mg/kg in saline, used as a reference drug) or SP-8356 (50 mg/kg in saline) once daily for 4 weeks. Rosuvastatin is a hydroxymethylglutaryl coenzyme A reductase inhibitor that has been well known to reduce neointima formation in animal models [23–25]. Statins also reduce restenosis characterized by neointimal hyperplasia after coronary stent implantation in clinical studies [26, 27]. At doses of 1, 10, and 20 mg/kg, rosuvastatin was reported to inhibit neointimal hyperplasia [23–25]. At a dose of 80 mg/kg, simvastatin has been known to be toxic to VSMCs [28], and recently it was reported that 20 mg/kg of rosuvastatin is equivalent to 80 mg/kg of simavastatin [29]. Therefore, we treated rats with 10 mg/kg of rosuvastatin. The in vivo therapeutic dose of SP-8356 corresponding to in vitro dose was determined based on our previous pharmacokinetic-pharmacodynamic relationship study [30].

### Pulse wave velocity (PWV) measurement

After 4 weeks of drug administration, PWV was measured using an ultrasound Doppler system (iU22, Philips Ultrasound, Bothell, WA, USA). Electrocardiography limb electrodes were placed and the Doppler probe was located parallel to the blood flow of the right carotid and left iliac arteries. The electrocardiography and Doppler signals were recorded simultaneously; the distance and time were defined as described previously [31]. Distance was measured between the site probe points over the carotid and iliac arteries, and the time was measured between the R peak waves of the electrocardiogram to the foot of the carotid or iliac wave signals. The times were averaged over three consecutive electrocardiography cycles. PWV (m/s) was calculated as:

$$ {\text{PWV }}\left( {{\text{m}}/{\text{s}}} \right) = \frac{\text{Distance from  carotid to iliac artery}}{{{\text{Time }}\left( {{\text{R peak point to iliac foot}} - {\text{R peak point to carotid foot}}} \right)}} $$

### Blood pressure

Blood pressure was measured using a tail-cuff method (ML125, Powerlab, AD Instruments, Castle Hill, NSW, Australia). Rats were placed in a chamber pre-heated to 35 °C for 10 min and moved to plastic restrainers. To obtain accurate and reliable blood pressure result, rats were handled gently and not forced to enter plastic restrainer. Rats remained stable and unperturbed during the measurement period. A cuff with a pneumatic sensor was applied onto the tail and blood pressure was measured, with the results averaged from three consecutive recordings.

### Histopathology

All rats survived during the study period and were sacrificed by carbon dioxide inhalation after 4 weeks of drug administration. The common carotid arteries were fixed with 4% paraformaldehyde and preserved in 30% sucrose solution. The tissue was embedded in optimal cutting temperature compound (Scigen Scientific, Gardena, CA, USA). Axial sections of 4 μm thickness were cut with a cryostat microtome (Leica CM 3050 S, Leica Microsystems). Serial sections were obtained down-stream of the bifurcation of the external and internal carotid arteries. In accordance with the guidelines for experimental study of vessel by the American Heart Association [32], lesions were analyzed in a blinded manner and quantified as an average of 6 serial sections, each 100 μm apart from each other. The sections were stained with hematoxylin and eosin and evaluated using an upright light microscope (BX51, Olympus, Tokyo, Japan). The neointimal area was defined as the area between the luminal circumference and the internal elastic lamina. The media area was defined as the area between the internal and external elastic lamina. The neointima/media ratio was defined as the area of the neointima divided by the area of the media.

### Immunohistochemistry

Sections were blocked for 60 min with 5% goat serum in phosphate-buffered saline containing 0.1% Triton X-100, and incubated at 4 °C with primary antibodies to α-smooth muscle-actin (α-SMA) (1:200 dilution, ab7817, Abcam, Cambridge, MA, USA), SM-MHC (1:200 dilution, ab683, Abcam, Cambridge, MA, USA), MMP-9 (1:200 dilution, AB19016, Merck Millipore, Billerica, MA, USA), and collagen type III (1:200 dilution, ab7778, Abcam, Cambridge, MA, USA). The sections were washed and incubated with the appropriate secondary antibody, Alexa Fluor 555-conjugated goat anti-mouse IgG (1:400 dilution, A21424, Invitrogen, Carlsbad, CA, USA) or Alexa Fluor 488-conjugated goat anti-rabbit IgG (1:400 dilution, A11034, Invitrogen, Carlsbad, CA, USA), followed by counterstaining of the nuclei with 4′, 6-diamidino-2-phenylindole (DAPI). All images were obtained using a confocal microscope (LSM800, Carl Zeiss, Oberkochen, Germany).

Molecular expression intensity in the neointimal area was analyzed using Image J version 1.45 s open-source software (NIH Image, Bethesda, MD, USA). A region of interest (ROI) was drawn on the neointimal area and the integrated density of pixels in the ROI was calculated. The intensity/area ratio was calculated as the integrated density divided by the area of the neointima. Unstained and secondary antibody stained control images of neointimal hyperplasia were shown in Additional file 1: Figure S1.

### Statistical analysis

All data were presented as the mean ± standard deviation of at least three independent experiments. Multiple groups were compared by one-way analysis of variance (ANOVA) followed by post hoc Tukey’s test and two groups were compared by Student’s *t* test. All statistical analyses were performed using SPSS version 17.0 software (SPSS Inc, Chicago, IL, USA), with a *p*-value < 0.05 considered statistically significant.

## Results

### SP-8356 disrupts the dimerization of CD147

**Fig. 1:**
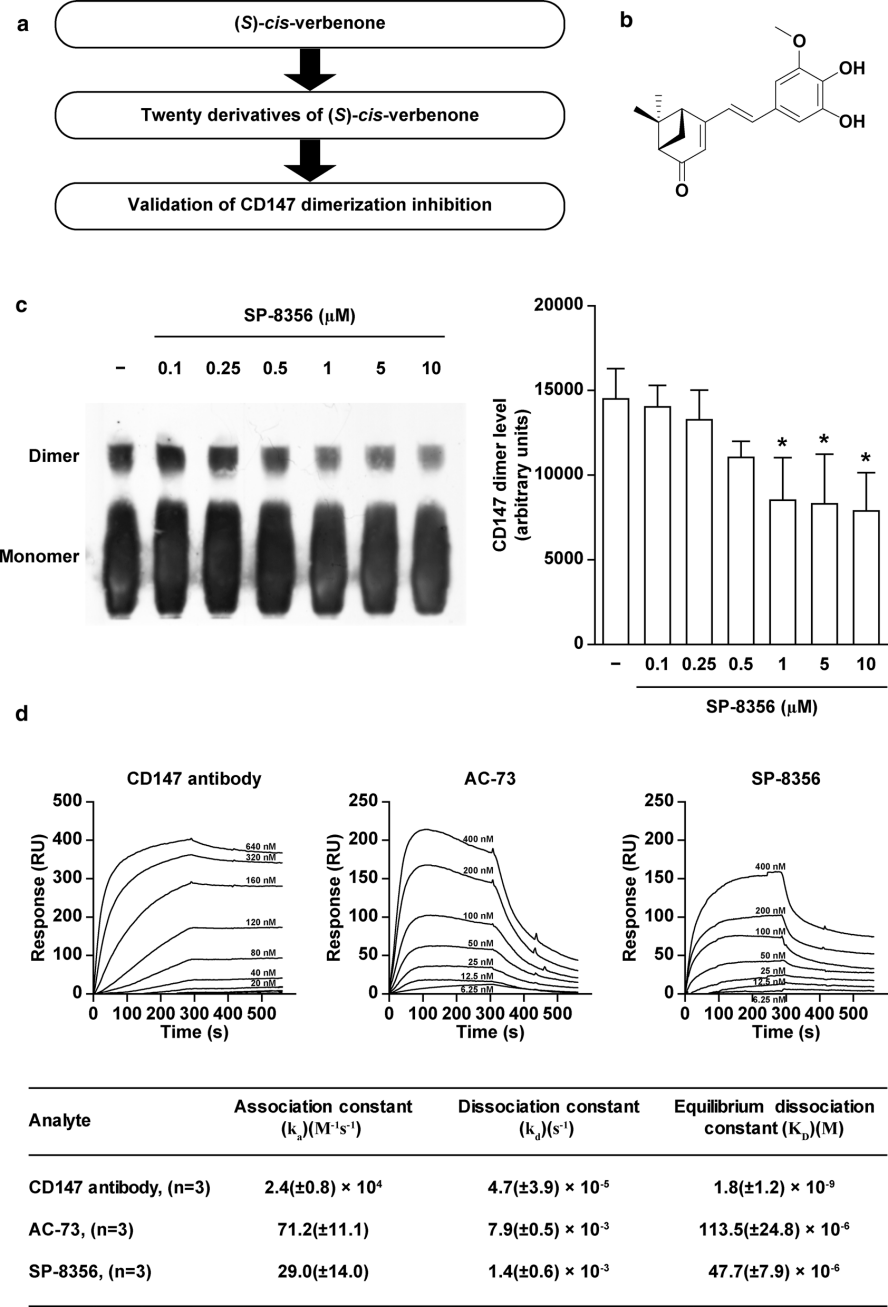
Discovery and activity of SP-8356. **a** Screening strategy. **b** Structure of SP-8356. **c** Representative image and quantitative analysis of CD147 dimerization. Data are presented as the mean ± standard deviation (SD) of three independent experiments. ^*^p < 0.05 vs. vehicle. **d** Surface plasmon resonance (SPR) assay of anti-CD147 antibody, AC-73, and SP-8356 with CD147

Dimerization of CD147 induces MMP expression and activation [14]. To identify compounds that could disrupt CD147 dimerization, we screened our in-house chemical library [15], finding that SP-8356 inhibited CD147 dimerization in a concentration-dependent manner (Fig. 1a–c).

### SP-8356 binds to CD147

Surface plasmon resonance (SPR) is an optical technique utilized for detecting molecular interactions [33]. We have utilized SPR to study the specific binding of SP-8356 with CD147. As shown in Fig. 1d, SP-8356 exhibited 3 times lower equilibrium dissociation constant (i.e., higher affinity) than that of AC-73, a previously described CD147 inhibitor [18].

### SP-8356 enhances the contractile phenotype expression in cultured VSMCs

**Fig. 2:**
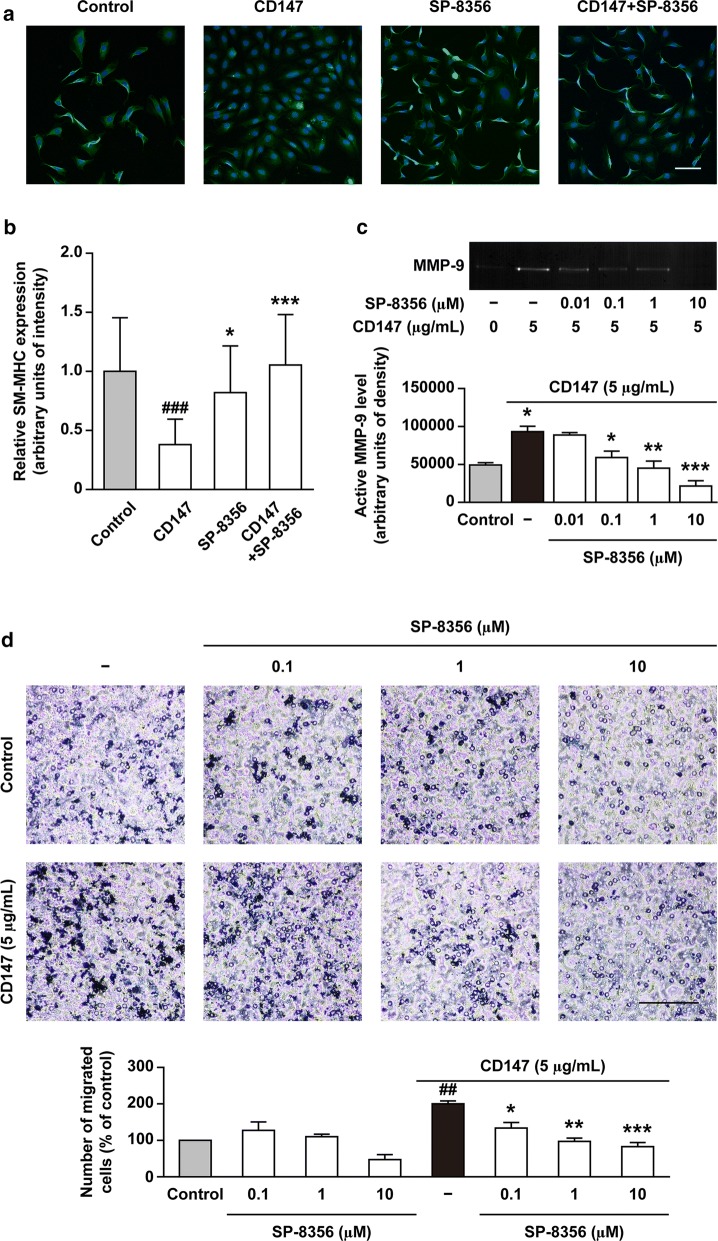
SP-8356 enhances the contractile phenotype expression and attenuates CD147-stimulated matrix metalloproteinase-9 (MMP-9) activation in vascular smooth muscle cells (VSMCs) and CD147-induced VSMC migration. **a** Representative images of immunocytochemistry and **b** quantitative analysis of relative smooth muscle myosin heavy chain (SM-MHC) expression. Scale bar, 100 μm. Magnification, × 100. Data are presented as the mean ± SD of three independent experiments. ^###^ *p* < 0.001 vs. control without CD147 treatment. ^*^p < 0.05, ^***^p < 0.001 vs. CD147 treatment alone. **c** Representative images of gelatin zymography and quantitative analysis of active MMP-9 level. Data are presented as the mean ± SD of three independent experiments. ^*^p < 0.05, ^**^p < 0.01, ^***^p < 0.001 vs. control. **d** Representative image of VSMC migration and quantitative analysis. Scale bar, 100 μm. Magnification, × 100. Data are presented as the mean ± SD of three independent experiments. ^##^ *p* < 0.01 vs. control without CD147 stimulation. ^*^p < 0.05, ^**^p < 0.01, ^***^p < 0.001 vs. CD147 stimulation alone

As shown in Fig. 2a, b, VSMCs cultured in serum-deprived condition showed increased expression of SM-MHC, which was reduced by treatment with recombinant CD147. SP-8356 reversed the recombinant CD147-induced reduction of SM-MHC expression.

### SP-8356 reduces CD147-evoked MMP and migratory activity in cultured VSMCs

A10 cells are undifferentiated VSMCs that differ from neonatal but show significant similarity to neointimal cells [34]. Thus, A10 cells have been commonly used as a model of synthetic VSMCs in neointimal hyperplasia. Gelatin zymography showed that treatment with recombinant CD147 increased MMP-9 activity in cultured VSMCs (Fig. 2c), which was inhibited by SP-8356 in a concentration dependent manner. Matrigel that is composed of basement membrane extracts has widespread use in cell migration assays [35]. Thus, as MMP-9 up-regulation is well known to promote VSMC migration [8, 9], we further performed Matrigel assays to test whether SP-8356 inhibits VSMC migration. In Matrigel assays, treatment with recombinant CD147 stimulated the migration of VSMCs (Fig. 2d), which was inhibited by SP-8356 in a concentration dependent manner.

### SP-8356 attenuates neointimal hyperplasia and improves arterial stiffness

**Fig. 3:**
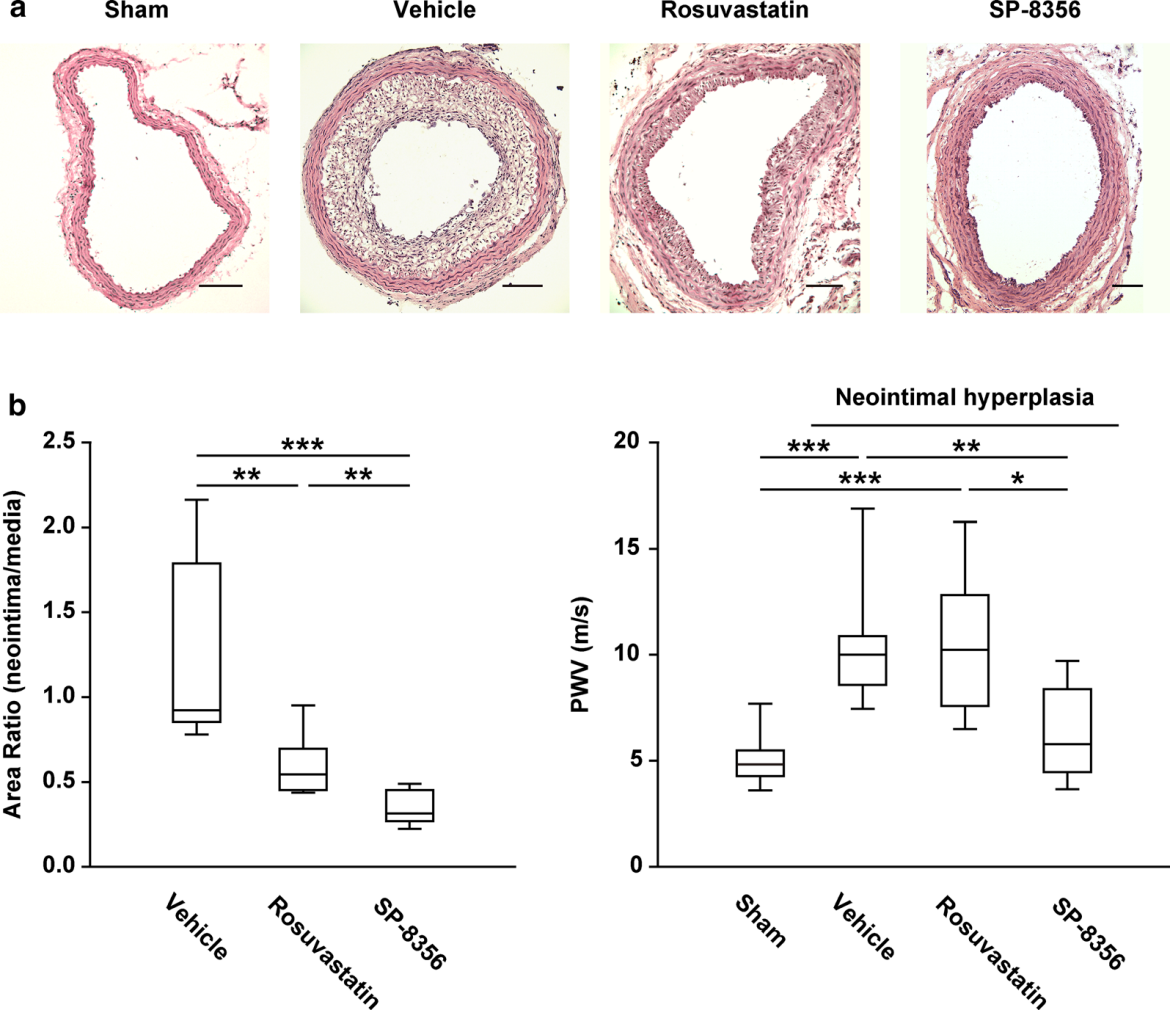
SP-8356 inhibits neointimal hyperplasia and prevents arterial stiffness. **a** Representative cross-sections of carotid arteries stained with hematoxylin–eosin (H&E). Scale bar, 100 μm. Magnification, × 100. **b** Ratios of neointima and media areas. Vehicle (*n* = 9), rosuvastatin (*n* = 6), and SP-8356 (*n* = 9). Data are presented as a box plot ± range. ^**^p < 0.01, ^***^p < 0.001. **c** Arterial stiffness was assessed by measuring pulse wave velocity (PWV). Normal (*n* = 15), vehicle (*n* = 9), rosuvastatin (*n* = 6), and SP-8356 (*n* = 6). Data are presented as box plot ± range. ^*^p < 0.05, ^**^p < 0.01, ^***^p < 0.001

While the normal artery presented little intima, the affected right carotid artery presented prominent luminal narrowing with neointimal hyperplasia (Fig. 3a). Both SP-8356 and rosuvastatin reduced neointimal hyperplasia (Fig. 3a). When neointimal hyperplasia was measured as the ratio of neointima to media, however, we found that SP-8356 reduced neointimal hyperplasia to a greater extent than rosuvastatin (Fig. 3b). Arterial stiffness assessed by measuring PWV was higher in rats with neointimal hyperplasia than in normal rats (Fig. 3c). SP-8356 reduced the aggravated arterial stiffness in rats fed a high fat/vitamin D diet (Fig. 3c). Despite its suppression of neointimal hyperplasia, however, rosuvastatin did not reduce the PWV elevated by neointimal hyperplasia (Fig. 3c).

There were no statistically significant differences among the normal, vehicle, and SP-8356 groups in systolic (128.39 ± 8.38 mmHg vs. 137.29 ± 8.38 mmHg vs. 134.33 ± 11.17 mmHg, *p* = 0.091) and diastolic (81.85 ± 8.46 mmHg vs. 87.17 ± 9.68 mmHg vs. 87.58 ± 10.92 mmHg, *p* = 0.448) blood pressure. These findings indicate that arterial stiffness was not influenced by blood pressure during or after SP-8356 treatment or experimental procedures.

### SP-8356 reduces MMP-9 expression and synthetic VSMC activity in neointima

**Fig. 4:**
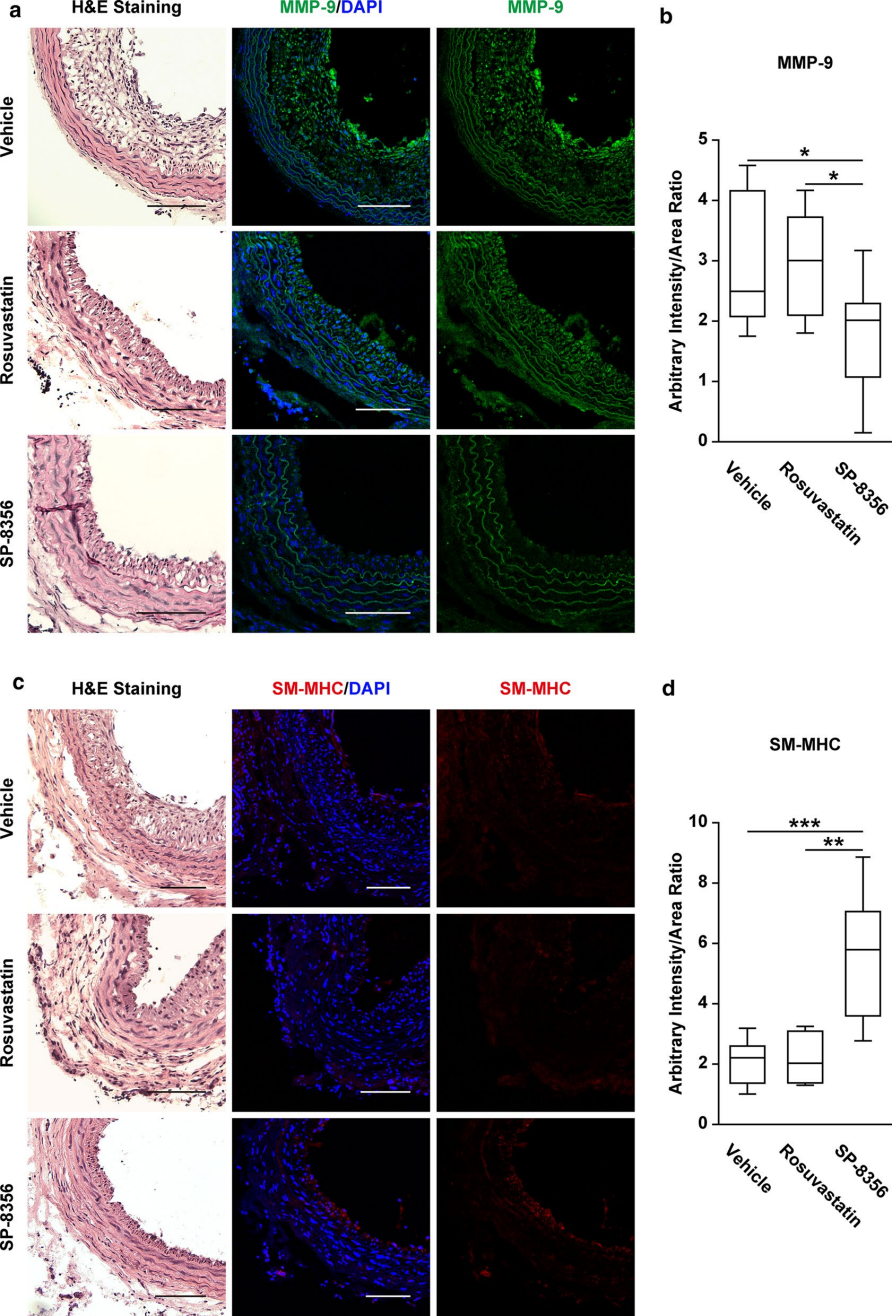
SP-8356 reduces MMP-9 expression and enhances SM-MHC. **a** Representative image of MMP-9 expression. Vehicle (*n* = 9), rosuvastatin (*n* = 6) and SP-8356 (*n* = 9). The nuclei were stained with DAPI. Scale bars, 100 μm. Magnification, × 100. **b** Quantification of MMP-9 expression. Data are presented as box plot ± range. ^*^p < 0.05. **c** Representative image of SM-MHC expression. Vehicle (*n* = 9), rosuvastatin (*n* = 6) and SP-8356 (*n* = 9). The nuclei were stained with DAPI. Scale bars, 100 µm. Magnification, × 100. **d** Quantification of SM-MHC expression. Data are presented as box plot ± range. ^**^p < 0.01, ^***^p < 0.001

**Fig. 5:**
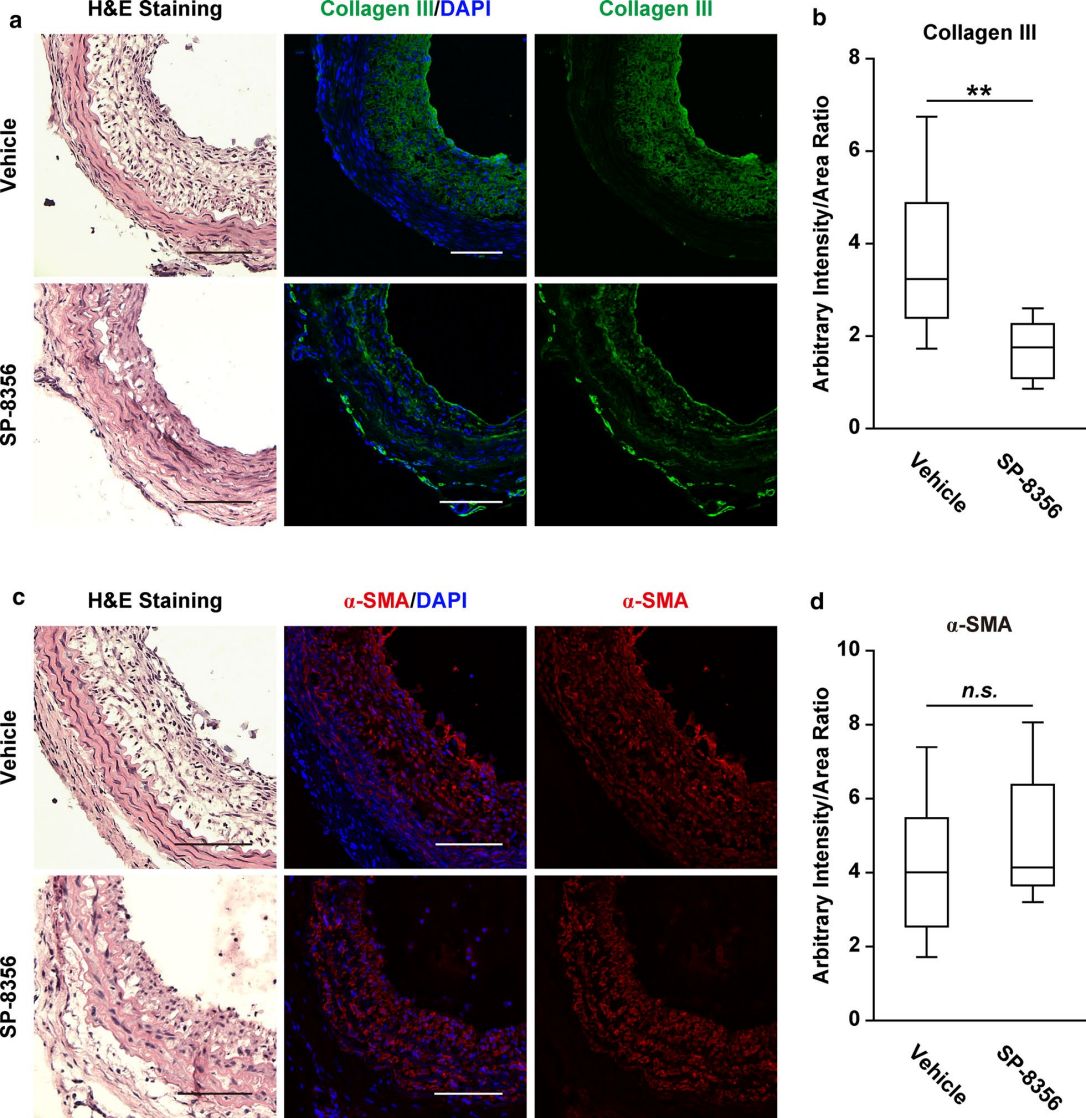
Down-regulation of collagen type III by SP-8356. **a** Representative image of collagen type III expression. Vehicle (*n* = 9) and SP-8356 (*n* = 9). Scale bars, 100 μm. Magnification, × 100. **b** Quantification of collagen type III expression. Data are presented as box plot ± range. ^**^p < 0.01. **c** Representative image of α-smooth muscle-actin (α-SMA) expression. Vehicle (*n* = 9) and SP-8356 (*n* = 9). The nuclei were stained with DAPI. Scale bars, 100 μm. Magnification, × 100. (D) Quantification of α-SMA expression. Data are presented as box plot ± range. n.s.: not significant

MMP-9 was abundantly expressed in proliferative neointima, which was significantly reduced by SP-8356 (Fig. 4a, b). In contrast, rosuvastatin treatment had no effect on MMP-9 expression (Fig. 4a, b). Because MMP-9 promotes synthetic VSMC characteristics [8], we studied the effects of SP-8356 treatment on VSMC characteristics. SP-8356 significantly increased the expression of SM-MHC (Fig. 4c, d) and reduced the expression of collagen type III (Fig. 5a, b), but did not significantly alter the expression of α-SMA (Fig. 5c, d). In contrast, rosuvastatin did not increase the expression of SM-MHC in affected carotid arteries (Fig. 4c, d).

## Discussion

CD147 is expressed in normal blood vessels, but its expression is higher under pathological conditions, during which CD147 may contribute to ECM remodeling by up-regulating MMP expression. The present study showed that a novel drug SP-8356 directly inhibited CD147 dimerization, suggesting that inhibition of the CD147-MMP pathway by SP-8356 may reduce neointimal hyperplasia in injured arteries and improve arterial stiffness.

MMP has been shown to promote VSMC migration, thereby contributing to neointimal hyperplasia [36]. Inhibition of MMP-9 expression by gene modulation was reported to reduce both VSMC migration and neointima formation [37, 38]. MMP also promotes synthetic properties of VSMCs [8], resulting in the deterioration of arterial stiffness [6]. In the present study, we found that SP-8356 elevated the level of SM-MHC and suppressed that of collagen type III, both of which could contribute to an improvement in arterial stiffness [6]. Similar to SP-8356, rosuvastatin was reported to reduce VSMC proliferation [23–25]. Unlike SP-8356, however, rosuvastatin did not improve arterial stiffness. The differing effects of SP-8356 and rosuvastatin on arterial stiffness may be due to differences in their regulation of MMP and SM-MHC expression. Our findings are in agreement with results showing that rosuvastatin inhibited neointimal hyperplasia via an MMP-9 independent mechanism [25, 39]. Furthermore, a high dose (80 mg/kg) of simvastatin was found to induce VSMC contractile dysfunction and to be toxic to VSMCs in rat models [28].

Although both SM-MHC and α-SMA reflect contractile properties of VSMCs [3], α-SMA is expressed by both synthetic and contractile VSMCs, whereas SM-MHC is expressed only by contractile VSMCs [2]. Thus, SM-MHC may be a more reliable marker of contractile VSMCs. Although SM-MHC expression in intimal VSMCs was found to be reduced during neointimal hyperplasia, α-SMA expression was preserved [2, 17, 40, 41]. Our experimental model also showed that SM-MHC expression was reduced, whereas α-SMA was unaltered, in neointima. Thus, the up-regulation of SM-MHC expression may contribute to the preservation of arterial elasticity in rats treated with SP-8356.

VSMC plasticity plays crucial roles in in-stent restenosis as well as atherosclerosis [4]. Revascularization by angioplasty/stenting is a common and effective treatment to restore blood flow in narrowed or blocked atherosclerotic lesions. In-stent restenosis, however, is a frequent complication of stent implantation, occurring in 15–60% of patients [42], and is thought to result from neointimal hyperplasia caused by synthetic VSMC activation. Thus, synthetic VSMCs are attractive therapeutic targets to inhibit neointimal proliferation after tenting [4]. In-stent restenosis may be prevented by implantation of drug-eluting-stents (DES) containing anti-proliferative drugs such as sirolimus and paclitaxel. These drugs inhibit the proliferation of VSMCs by targeting the mechanistic target of rapamycin (mTOR) or microtubule assembly [43, 44]. However, these DESs can elicit late thrombosis by the attenuation of re-endothelization through endothelial toxicity [22, 45] and even increase the long-term risk of death compared with bare-metal stents after 6 months [45]. There is a pressing need for new drugs that inhibit restenosis for successful revascularization without late thrombosis. The results of the present study suggest that SP-8356 may be a promising new drug to inhibit in-stent restenosis.

Neointimal hyperplasia with VSMC activation involves complex multifactorial pathophysiology including inflammation and oxidative stress [46, 47]. MMP-9 modulation via its interaction with CD147 may not be the sole explanation for the vasoprotective effect of SP-8356. SP-8356 was designed to function as a multi-target directed drug with anti-inflammatory and anti-oxidative properties [15, 48]. Thus, the pleiotropic effects of SP-8356 may also contribute to the vasoprotective effects of SP-8356.

This study used a rat model of partial carotid artery ligation to evaluate the arteries with pathological remodeling. This model closely resembles the pathophysiologic hemodynamic features of atherosclerosis in humans, including low shear stress, which contribute to the formation of neointimal hyperplasia [20]. This model is therefore suitable for the evaluation of vascular remodeling disorders, in which synthetic VSMC migration is the main pathologic feature. This rat model, however, has limitations, including its inability to mimic the plaque vulnerability, rupture, and thrombosis found in advanced atherosclerotic lesions [20]. Other models of advanced atherosclerotic lesions, such as apolipoprotein E knock-out mice, are needed to fully assess the anti-atherosclerotic activities of SP-8356.

Although SP-8356 effectively reduced MMP activity by inhibiting CD147 dimerization in in vitro experimental methods, it may also have other off-target binding effects in addition to CD147. For example, CD147 increases MMP activity through binding with cyclophilin A [10], which may also be inhibited by SP-8356. Therefore, further studies on the target profile of SP-8356 are needed.

## Conclusions

This study provides strong evidence that SP-8356 can inhibit neointimal hyperplasia and improve arterial stiffness in a rat model of partial carotid artery ligation. Although its full mechanism remains to be determined, SP-8356 exhibits vasoprotective effects, probably by disrupting CD147 dimerization and thereby suppressing MMP-9 expression. SP-8356 may be a promising therapeutic drug for the treatment of vascular remodeling disorders.

## Supplementary information

**Additional file 1: Figure S1.** Unstained and secondary antibody stained control images of neointimal hyperplasia. There were no specific signals in both neointima and media. In unstained image, autofluorescence was significantly observed only in elastic lamina. In secondary antibody stained images, 488 nm signals were observed in elastic lamina and 555 nm signals in tissue debris and the boundary of neointima. Scale bars, 100 μm. Magnification, × 100.

## Data Availability

The datasets used and/or analysed during the current study are available from the corresponding author on reasonable request.

## Abbreviations

CD147: cluster of differentiation 147
VSMC: vascular smooth muscle cell
ECM: extracellular matrix
MMP: matrix metalloproteinase
SPR: surface plasmon resonance
PWV: pulse wave velocity
α-SMA: α-smooth muscle-actin
SM-MHC: smooth muscle myosin heavy chain
DES: drug-eluting-stents
