# Supplementary figures and images for: A novel CD147 inhibitor, SP-8356, reduces neointimal hyperplasia and arterial stiffness in a rat model of partial carotid artery ligation
Source: J Transl Med. 2019 Aug 20;17:274. doi: 10.1186/s12967-019-2024-y (PMC6700999; doi:10.1186/s12967-019-2024-y)

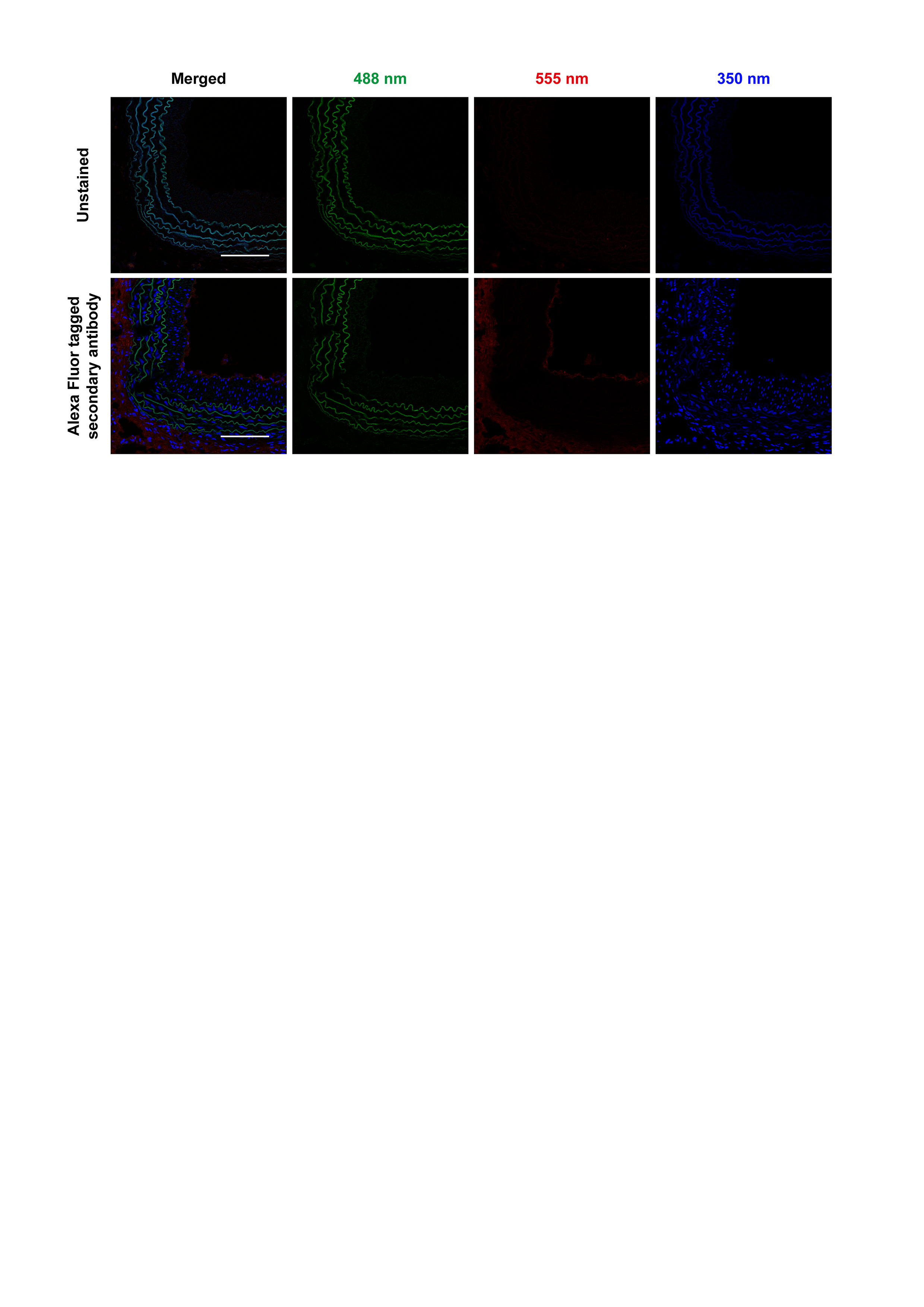

Supplement: Supplementary file 1 — Additional file 1: Figure S1. Unstained and secondary antibody stained control images of neointimal hyperplasia. There were no specific signals in both neointima and media. In unstained image, autofluorescence was significantly observed only in elastic lamina. In secondary antibody stained images, 488 nm signals were observed in elastic lamina and 555 nm signals in tissue debris and the boundary of neointima. Scale bars, 100 μm. Magnification, × 100. [file 12967_2019_2024_MOESM1_ESM.tif]
